# PZT Composite Film Preparation and Characterization Using a Method of Sol-Gel and Electrohydrodynamic Jet Printing

**DOI:** 10.3390/mi14050918

**Published:** 2023-04-24

**Authors:** Yan Cui, Hao Yu, Zeshan Abbas, Zixiang Wang, Lunxiang Wang, Dazhi Wang

**Affiliations:** 1 Key Laboratory of Precision and Special Processing of Ministry of Education, Dalian University of Technology, Dalian 116024, China; yuhao980828@163.com (H.Y.); zixiang@mail.dlut.edu.cn (Z.W.); 13156898415@163.com (L.W.); d.wang@dlut.edu.cn (D.W.); 2 Institute of Intelligent Manufacturing Technology, Shenzhen Polytechnic, Shenzhen 518055, China; hopenotout1214@mail.dlut.edu.cn; 3 Ningbo Institute of Dalian University of Technology, Ningbo 315000, China

**Keywords:** e-jet printing method, PZT thick film, Sol-gel method, PZT thin film, composite film

## Abstract

Lead zircon titanate (PZT) composite films were advantageously prepared by a novel hybrid method of sol-gel and electrohydrodynamic jet (E-jet) printing. PZT thin films with thicknesses of 362 nm, 725 nm and 1092 nm were prepared on Ti/Pt bottom electrode via Sol-gel method, and then the PZT thick films were printed on the base of the PZT thin films via E-jet printing to form PZT composite films. The physical structure and electrical properties of the PZT composite films were characterized. The experimental results showed that, compared with PZT thick films prepared via single E-jet printing method, PZT composite films had fewer micro-pore defects. Moreover, the better bonding with upper and lower electrodes and higher preferred orientation of crystals were examined. The piezoelectric properties, dielectric properties and leakage currents of the PZT composite films were obviously improved. The maximum piezoelectric constant of the PZT composite film with a thickness of 725 nm was 69.4 pC/N, the maximum relative dielectric constant was 827 and the leakage current was reduced to 1.5 × 10^−6^A at a test voltage of 200V. This hybrid method can be widely useful to print PZT composite films for the application of micro-nano devices.

## 1. Introduction

In recent times, the lead zircon titanate (PZT) material has been widely employed in piezoelectric sensors and actuators due to its prominent and high electromechanical coupling coefficient, piezoelectric constant and relative dielectric constant [1,2]. The principle of electromechanical conversion in PZT material is based on the piezoelectric effect. When PZT is subjected to external mechanical force, it accumulates opposite charges on its two surfaces, which is called the piezoelectric effect. When PZT is subjected to an external electric field, the PZT’s structure undergoes deformation, which is called the inverse piezoelectric effect [3].

Due to the wide application of PZT materials in the field of piezoelectric actuators and sensors, higher requirements have been placed on the structural size of PZT materials [4]. According to their thickness, PZT materials are usually divided into PZT bulk materials and PZT film materials. PZT bulk materials refer to those with a thickness greater than 100 μm, while PZT film materials refer to those with a thickness less than 100 μm. Moreover, PZT film materials can be further divided into PZT thin films and PZT thick films based on their thickness. PZT thick film (5 μm−100 μm) is a film-like material with a thickness between thin film and bulk material that offers the benefits of compact size, light weight, high piezoelectric driving force and a wide operating frequency range [5]. Therefore, with the ongoing development of current microelectronics technology, particularly MEMS technology and the preparation of PZT thick films and their functional devices (e.g., energy collectors, micro-actuators and high-resolution piezoelectric sensors), there have been extensive application opportunities and development space [6]. PZT thick film preparation methods include screen printing, air floatation deposition, electrojet printing and others that are very widely used by researchers [7,8,9,10,11]. The electrojet printing method is based on electrohydrodynamic jet deposition, which employs liquid fluid to generate a finely stable jet under the action of electric field force, self-gravity and viscous force. It ejects nano-droplets to be deposited on the surface of the substrate and carries out the deposition and shaping of the material with layer-by-layer accumulation [12].

Electrojet printing as a micro-nano scale additive manufacturing method offers benefits such as high molding precision, strong controllability and substrate adaptability, etc. [13,14,15]. Micro-pore flaws are common in PZT thick films due to its unique crystallization mechanism, resulting in a relatively high leakage current and poor dielectric characteristic. Wang et al. investigated the influence of the ball milling time of PZT suspension on PZT thick film [16,17]. PZT particle size decreased from 0.8 mm to 0.2 mm after 40 h of ball milling. Ball milling PZT sol and PZT powder produce stable slurry suited for printing and minimizes micropores between PZT thick films. Therefore, it enhances the performance of PZT thick films. The leakage current of the PZT thick films was made using this approach. However, it remains high.

This study suggests using the PZT thin film coating as a seed layer to improve the electrical characteristics of PZT thick film to alleviate the difficulties of PZT thick film [18]. The PZT thin film’s dense structure can reduce the leakage current of PZT thick film. The PZT thin film fabrication techniques primarily comprise ultrasonic spray combustion synthesis, magnetron sputtering, diffusion and Sol-gel [19]. The Sol-gel method is easier to study the influence of PZT thin film on PZT thick film because of its simple process, low cost, controllable Zr/Ti composition ratio and compatibility with MEMS/NEMS technology [6,20,21,22]. In addition, the film prepared by this method has compact structure, uniform physical and chemical properties and large size film forming ability, which has been widely studied domestically and internationally. Therefore, this work adopts the Sol-gel method to prepare PZT thin film.

In the present study, PZT composite films were prepared by productive hybrid processing: the Sol-gel and E-jet printing method. PZT composite films with different thicknesses of the PZT thin films and a certain thickness of the PZT thick films were prepared. The physical and electrical properties of PZT composite films with different thicknesses were studied.

## 2. Experimental Procedure

**Figure 1:**
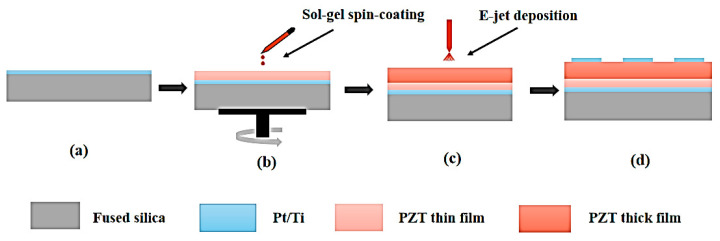
Illustration of the schematic of fabrication route of the PZT composite films (**a**) preparation of Ti/Pt bottom electrode (**b**) PZT thin films formation by spin coating (**c**) PZT thick films by E-jet deposition (**d**) preparation of Ti/Pt top electrode.

The PZT composite film was designed on fused silica substrates. The structure consisted of five parts: the fused silica substrate, Ti/Pt metal bottom electrode, PZT thin film, PZT thick film and Ti/Pt metal top electrode. This fabrication process is depicted in Figure 1. The Ti/Pt metal bottom electrode was deposited on a fused silica substrate via magnetron sputtering, as shown in Figure 1a. Figure 1b shows different thicknesses of PZT thin films that were produced on the bottom electrode using the Sol-gel process. The thicknesses of the PZT thin films were measured using a J.A. Woollam spectral ellipsometer (M-2000, Woollam, Lincoln, NE, USA), and the thicknesses of the PZT films with 6, 12 and 18 layers were about 362, 725 and 1092 nm, respectively. Figure 1c indicates PZT thick films with thicknesses of 6 μm that were deposited on PZT thin films by E-jet printing. Figure 1d depicts a Ti/Pt metal top electrode that was deposited on the PZT thick films. To mitigate the problem of lateral corrosion when the top electrode was corroded, the lift-off process was used to fabricate the top electrodes. First, the photoresist was spin-coated on the PZT film and then pre-baked at 85 °C for 30 min to harden the photoresist film. Then, the photoresist was exposed by a German Karl Suss lithography machine (SUSS, Garching, Germany) to achieve the purpose of pattern transfer, which was followed by the patterning of the photoresist by the developer. Second, 200 nm thick Pt and 50 nm thick Ti were deposited onto the patterned photoresist. Finally, the samples were soaked in acetone for 30 min to remove the photoresist, and the desired top electrode pattern was obtained.

### 2.1. Preparation of PZT Thin Films

The precursor solution of the PZT sol was composed of zirconium nitrate (Zr(NO_3_)_4_·5H_2_O), lead acetate (Pb(CH_3_COO)_2_·3H_2_O) and butyl titanate (C1_6_H_36_O_4_Ti). The solvent was ethylene glycol methyl ether (C_3_H_8_O_2_) and acetylacetone (C_5_H_8_O_2_), where the mass ratio of zirconium nitrate to lead acetate was maintained at 1:2. The stable PZT precursor sol was obtained by mixing and stirring the precursor solution and standing for 24 h. First, the PZT sol was uniformly dripped onto the substrate and then spin-coated for 6 sec at a rotational speed of 600 r/min using a glue throwing machine (KW-4A). Equally, it was spin-coated for 30 sec again at a rotational speed of 3000 r/min. Second, the sample was heated on a conventional muffle furnace at a temperature of 180 °C for 5 min to remove water from the PZT thin film. Ultimately, the sample was heat treated in a muffle furnace at 350 ℃ for 5 min to decompose the organic matter in the film. The spin coating and heat treatment processes were repeated for odd layers. When the PZT thin film was an even layer, the substrate was sintered in a muffle furnace at 600 ℃ for 8 min. Therefore, the PZT thin film crystallized fully and formed perovskite structure in the system [14]. The spin coating and heat treatment processes were repeated in turn to reach the required thickness.

### 2.2. Preparation of PZT Thick Films

The PZT thick film was prepared based on the PZT thin film’s preparation. The PZT suspension, which consisted of PZT powder and PZT sol, was used as slurry in a thick film deposition experiment. The PZT suspension had higher viscosity and better stability in the E-jet deposition process compared with those of the PZT sol. The PZT solid was prepared: titanium isopropoxide (Ti_4_ (OCH_3_)_16_) and zirconium n-propoxide (C_12_H_28_O_4_Zr) were mixed and stirred under N_2_ atmosphere for 5 min and the stirring speed was 360 r/min. The glacial acetic acid (CH_3_COOH) and n-propanol (C_3_H_8_O) were added and stirred for 5 min. When the lead acetate was completely dissolved in the solution, n-propanol and glacial acetic acid were added and stirred for 4 h to obtain PZT sol. The metal ion ratio of Pb:Zr:Ti in the prepared PZT sol was 1.10:0.48:0.52. Then, PZT powder, PZT sol and dispersant (Ken-ReactLica38) were uniformly mixed. Finally, the ball milled for 40 h in a ball mill was placed to form the PZT suspension.

**Figure 2:**
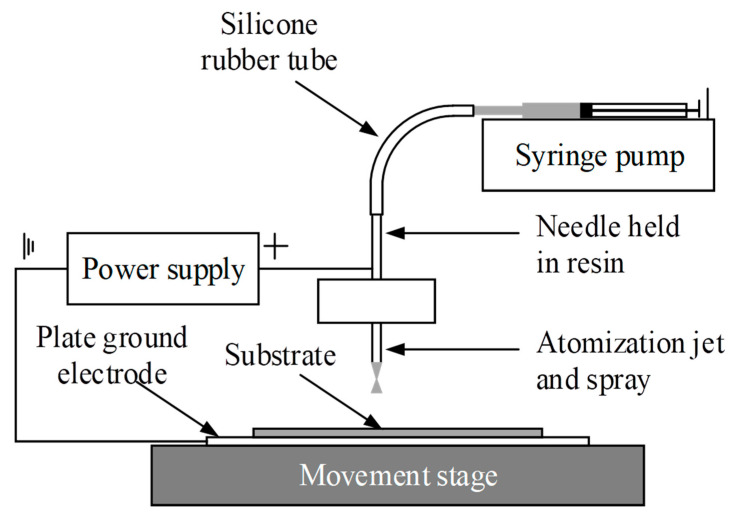
The schematic diagram of E-jet deposition equipment set-up.

The PZT thick film was fabricated by the E-jet deposition device. The E-jet deposition apparatus is shown in Figure 2, which mainly comprised an E-Jet printing needle, a computer controlled X–Y movement stage, a high power supply and a syringe pump. This E-Jet printing device was developed in our lab. The needle was connected to the high voltage power supply (Tianjin DongWen High Voltage Power Supply Co., Ltd., Tianjin, China), which was used to provide an electric field between the needle and the ground electrode. The inlet of the needle was connected to the syringe pump (LSP01-2A, Longer-Pump, Baoding, China), which provided the hydrodynamic force to push the PZT slurry to the outlet of the needle. In this work, the needle had an inner and outer diameter of 0.2 mm and 0.7 mm, respectively. The E-Jet printing process could be observed in real-time by using the microscopic vision system. The E-jet deposition parameters were as follows: the flow rate of the PZT suspension was 1.66 × 10^−10^ m^3^·s^−1^, the height between the spray needle and the substrate was set at 4 mm. The applied DC voltage was used at 3.6–4.5 kV. The spray needle moved alternately in the X and Y directions at a speed of 35 mm/s to achieve layer-by-layer deposition of materials. The deposition path spacing of the PZT suspension was set to 1.4 mm to ensure the uniformity of the monolayer deposition thickness. The PZT thick film was dried in an oven at 200 ℃, which ensured that one layer of PZT thick film was deposited each time. Then, the PZT thick film was annealed in an oven at 350 ℃. A series of experiments were performed using all the samples to achieve the required thickness of the printed structures.

## 3. Results and Discussion

### 3.1. Physical Structure Analysis of PZT Composite Films

**Figure 3:**
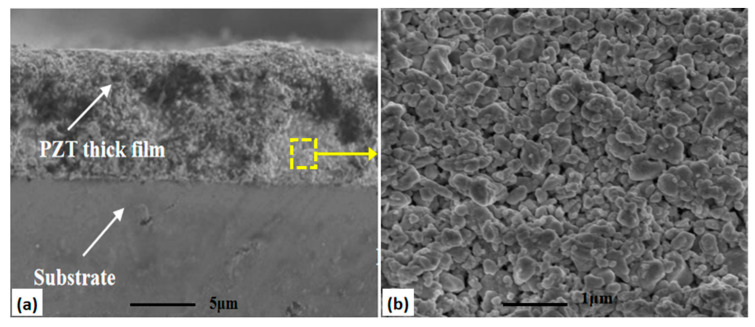
The cross-section SEM image of the PZT thick film structures: (**a**) cross-section of the PZT thick film and its high-magnification image (**b**).

**Figure 4:**
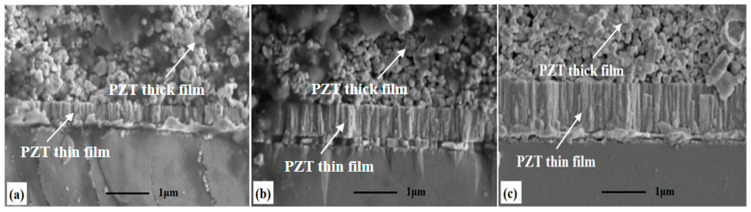
The cross-section SEM images of the PZT composite films with thin film thicknesses of (**a**) 362 nm, (**b**) 765 nm and (**c**) 1095 nm.

Scanning electron microscopy (SEM, SU8200, Hitachi High-Technologies, Tokyo, Japan) was used to characterize the cross-sectional morphology of all samples. The cross-sectional structure test result of the PZT thick film sample is shown in Figure 3a,b. The PZT thick film prepared by E-jet printing was not fully dense inside and had micro-pore defects inside. The cross-sectional structure test results of the PZT composite film samples are shown in Figure 4. Figure 4a–c illustrates the cross-sectional views of PZT composite films with thin film thicknesses of 362 nm, 725 nm and 1092 nm, respectively.

The cross-sectional morphology of the PZT thin films presented uniform and dense columnar perovskite structures. The PZT thin films maintained flat and good contact interface conditions with the bottom electrodes and PZT thick films. The thin PZT film had no effect on the thick film in the PZT composite film. The PZT thick film was prepared based on the PZT thin film where obvious defects such as cracks and pores were not examined. The cross-sectional morphology of each film layer kept the compact columnar structure very bright and decent. The cross-sectional structure test results showed that the PZT composite film had good crystallization condition. Furthermore, there were good contact and bonding conditions between the electrode and each layer [15].

**Figure 5:**
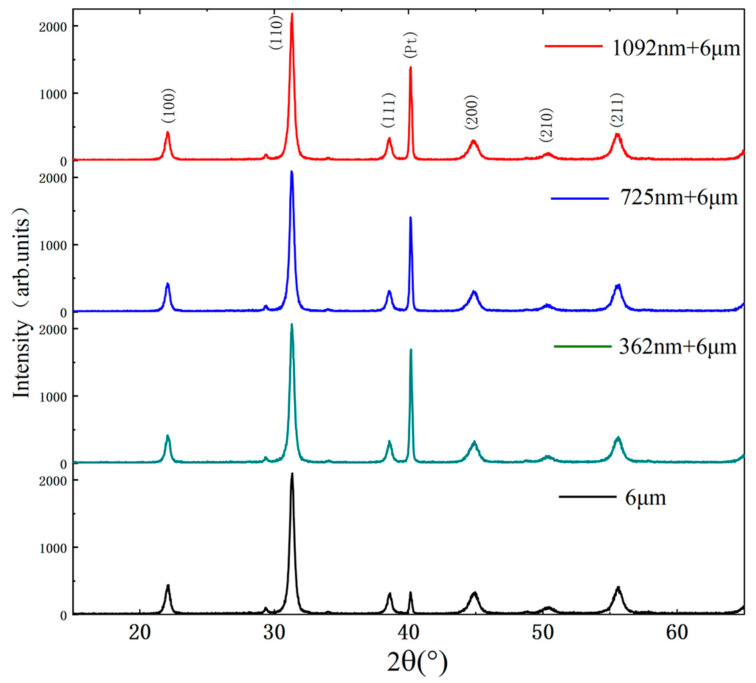
The XRD spectra of PZT composite films and PZT thick film with different thicknesses.

The phase composition was examined by XRD using a scanning rate of 0.04°/s in the range of 15–65°, and the θ−2θ scanning mode was selected. An X-ray diffractometer (XRD, D8 Bruke and Zeiss, Karlsruhe, Germany) was used to characterize the crystal orientation of the different samples, and the results are shown in Figure 5. It can be seen from Figure 5 that all samples are composed of perovskite phases without secondary phases such as pyrochlore phases Pb_2_Ti_2_O_7_. Therefore, it can be seen that there are six standard PZT peaks via comparing the diffraction pattern with the standard PDF card, which are (100), (110), (111), (200), (210) and (211), respectively. The preferred and suitable orientation of the samples is (110). The formula for calculating the degree of preferred (110) and good (α) orientation of the PZT composite film is as follows [23]:

(1) $$\alpha =\frac{I(110)}{I(100)+I(110)+I(111)}\times 100\%$$

where I _(100)_, I _(110)_ and I _(111)_ are the intensity of peak (100), peak (110) and peak (111), respectively. The PZT composite films with 362 nm, 725 nm and 1092 nm thicknesses (110) had the preferred orientation (α) of 73.42%, 73.85% and 74.12%, respectively. The PZT thick films had a preferred orientation α of 73.38%. The PZT thick films prepared by E-jet printing were crystallized with PZT powder particles in suspension as the core. The PZT thin films prepared by the Sol-gel method were crystallized layer by layer on the bottom electrode. All of the samples had a relatively high preferred orientation (α) of (110).

### 3.2. Electrical Properties Analysis of PZT Composite Films

The electrical properties of the PZT composite films mainly include leakage current characterization, piezoelectric classification and dielectric properties. Because the relative orientation of spontaneous polarization of the PZT thick film has no obvious law and the direction of spontaneous polarization is consistent after artificial polarization [24], polarization refers to heating PZT piezoelectric material above the Curie temperature and applying an electric field, maintaining it for a certain period of time, and then stopping the heating process. The electric field is removed when cooled below the Curie temperature. There are three important parameters in the polarization process: polarization field strength, polarization temperature and polarization time. The polarization conditions are related to the structure, composition and thickness of the PZT material. Therefore, the polarization process used in this study is as follows: PZT material was polarized for 20 min at a polarization field strength of 11 V/μm and a polarization temperature of 200 °C. Then, the polarization temperature was gradually reduced to 60 °C and the electric field was removed. The electric field intensity before the test of its electrical properties was evaluated.

**Figure 6:**
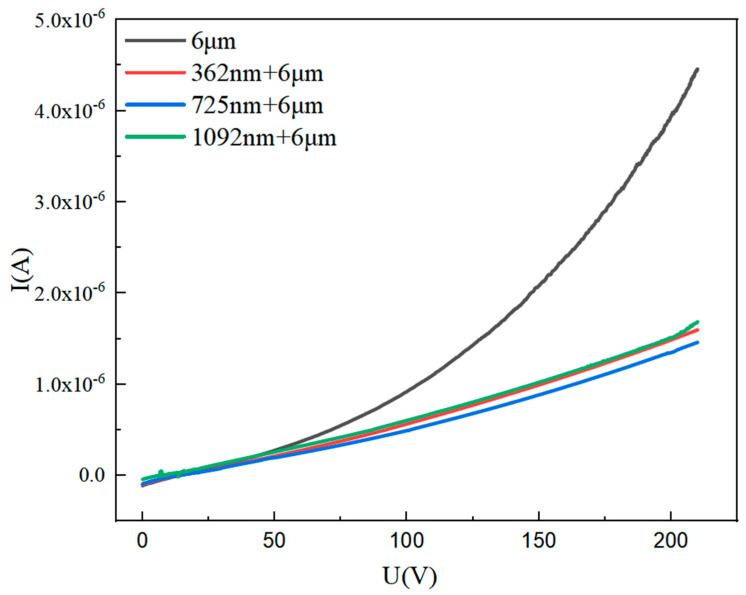
The leakage current curves of the PZT composite films and the PZT thick film with different thicknesses.

Leakage current tests of the PZT thick film and the PZT composite film with film thicknesses of 362 nm, 725 nm and 1092 nm were carried out using a semiconductor parameter tester (Keithley, 4200A-SCS, Cleveland, OH, USA). Figure 6 shows the leakage current results of all samples. It can be seen that the leakage current of the PZT thick film and the PZT composite films increases with the increase in the applied voltage. In the process of preparing the PZT thick film, there are usually some defects that lead to the incomplete insulation of the PZT thick film such as micro-pore defects, a number of conductive carriers and inconsistent film compactness. These factors lead to some free charges in the material and form weak leakage currents. It is better when the leakage current of the prepared PZT material is small since the existence of a leakage current cause the PZT thick film device to heat up and reduce the lifetime of the device. Therefore, reducing the sample leakage current is the key to improving the performance of PZT devices. The leakage current of the composite PZT film is significantly reduced compared with that of the thick PZT film. At 200V voltage, the leakage current of the PZT composite film decreases from 4 × 10^−6^A to 1.5 × 10^−6^A and the leakage current decreases by 62.5%. The dense structure of the PZT thin film limits the leakage current of the entire composite film. Inserting the thin PZT film as a series resistance layer can effectively reduce the leakage current of the thick PZT film.

The piezoelectric constant is a conversion coefficient that describes the ability of a piezoelectric material to convert mechanical energy into electrical energy or electrical energy into mechanical energy. It is an important parameter of piezoelectric materials. The higher the piezoelectric constant d_33_ of a piezoelectric film, the greater its sensitivity will be. According to the IEEE test standard, the common test methods for effective piezoelectric constants (d_33_) are the quasi-static and resonance methods. The quasi-static method has the advantages of simple operation, high reliability and a wide measurement range. Therefore, in this paper, we have selected the quasi-static electrical measurement method to measure the piezoelectric constant d_33_ of PZT films. The test of piezoelectric properties was carried out using a d_33_ quasi-static tester (LC2735A). The quasi-static tester consists of the test specimen, standard specimen and two capacitors. The working principle is to apply a low-frequency alternating force F to the test and standard specimens simultaneously through a shaker, with the force applied in the same direction as the polarization direction of the piezoelectric material. Due to the positive piezoelectric effect of the piezoelectric material, the test and standard specimens generate electrical signals, which are, respectively, conducted to the capacitors C1 and C2. The voltages V1 and V2 at the two ends of the capacitors are then measured by the circuit, and after calculation, the d_33_ of the test specimen can be obtained.

The particular test results of piezoelectric constant d_33_ of the PZT composite film are given in Table 1. The piezoelectric constants of PZT composite films with thicknesses of 362 nm, 725 nm and 1092 nm are 52.6 pC/N, 69.4 pC/N and 53.7 pC/N, respectively. The piezoelectric constants of PZT thick film is 48.8 pC/N. It can be perceived that the piezoelectric properties of the PZT composite films are further improved compared with the PZT thick film prepared by E-jet printing method. Additionally, the PZT thin film itself has certain piezoelectric properties. Further, the compact structure of PZT thin film can improve the overall electrical properties and, thus, enhance the piezoelectric properties. Consequently, the introduction of PZT thin film improves the piezoelectric properties of PZT thick film.

**Figure 7:**
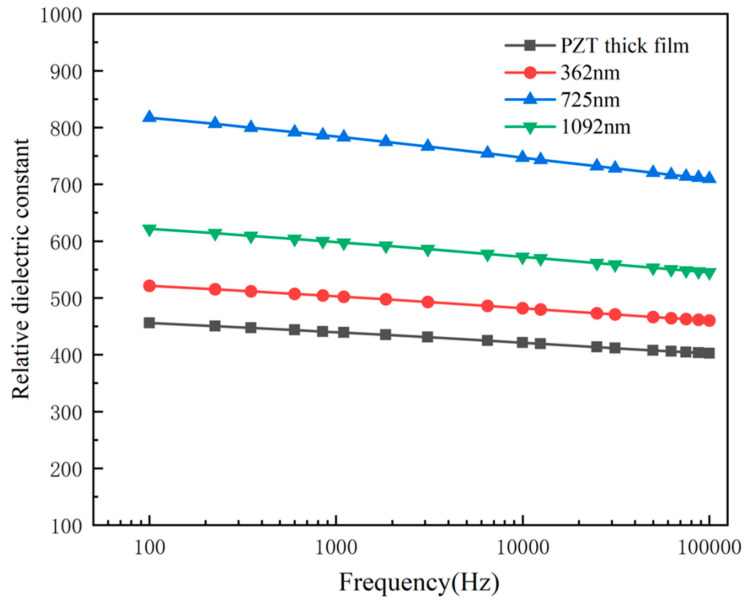
The relative dielectric constant of the PZT composite films and the PZT thick film with different thicknesses.

The dielectric properties of PZT materials refer to the storage properties of electric energy when they are placed in an electric field. Generally, the dielectric properties of PZT materials are characterized by dielectric constant and dielectric loss. However, the absolute dielectric constant of materials is relatively small. This work usually uses the relative dielectric constant to express the explanation. The calculation formula is as follows [25]:

(2) $$\varepsilon =\frac{C\cdot d}{A\varepsilon_{0}}$$

where *ε* represents the relative dielectric constant; *ε*_0_ represents the vacuum dielectric constant, and its value is 8.85 × 10^−12^ F/m. Therefore, *C* is a piezoelectric film capacitor, which is obtained via test with an impedance analyzer, and *d* is the thickness of piezoelectric film. Thus, *A* is the effective contact area of the piezoelectric film electrode. Test results of relative dielectric constants of all samples are shown in Figure 7. The maximum relative dielectric constants of the PZT thick film and the PZT composite films with thicknesses of 362 nm, 725 nm and 1092 nm are 459, 521, 827 and 623, respectively. It shows that the PZT composite films have better ability to store charge compared with the PZT thick film. The relative dielectric constant of the PZT composite films increases significantly, the maximum relative dielectric constant reaches 827 and the relative dielectric constant increases by 83%. When the leakage current of the PZT composite films is smaller, then the free charges in the PZT composite films are smaller. The fixed charge stored in PZT composite films is larger and the relative dielectric constant is larger. More impurities and external defects can be introduced into the preparation process as the thickness of the prepared PZT thin films increases. Likewise, the residual stresses of the thin films increase with increasing annealing crystallization time, which will affect the relative dielectric properties of the PZT composite films. It can be seen from Figure 7 that thinner or thicker PZT films will reduce the relative dielectric properties of the PZT composite films. Therefore, the relative dielectric properties of the PZT composite film are the best when the thickness of the PZT thin film is 725 nm.

**Figure 8:**
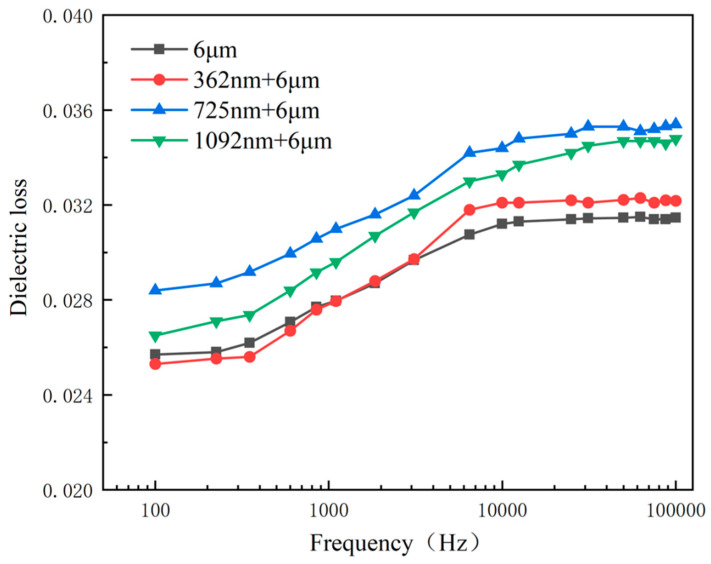
The dielectric loss of the PZT composite films and the PZT thick film with different thicknesses.

Test results of dielectric loss of samples are shown in Figure 8. Due to the increase in space charge and interface polarization contribution inside the PZT film, the dielectric loss of the PZT composite films were higher compared with the dielectric loss of the PZT thick film. The experimental results display that the dielectric loss of the four samples did not exceed 0.04. The fluctuation range analyzed was very small and the stability in the process of charge transfer and conversion was good.

## 4. Conclusions

In this paper, PZT composite films are successfully prepared based on a new hybrid process (Sol-gel and E-jet printing method). PZT composite films with different thicknesses of PZT thin films were studied. Simultaneously, the X-ray diffraction patterns, microstructures, leakage currents, dielectric and piezoelectric properties of all samples were investigated. The experimental results show that the PZT thin film and the PZT thick film were well bonded. The preparation process of the PZT thick film did not affect the structure of the prepared PZT thin film. The experimental approach achieved PZT composite film with a lower current flow and higher piezoelectric and dielectric properties compared with those of the PZT thick film. The maximum piezoelectric constant of the PZT composite film with a film thickness of 725 nm was 69.4 pC/N, and the maximum relative dielectric constant was 827, which are 83% higher than those of the single thick PZT film. Therefore, the leakage current decreased from 4 × 10^−6^A to 1.5 × 10^−6^A under a test voltage of 200V, which is a total percentage of 62.5%. Consequently, PZT composite films improve the electrical properties of the PZT material and have broad application prospects combined with M/NEMS technology.

## Figures and Tables

**Table 1:** Piezoelectric constant of PZT composite films with different film thicknesses.

Thickness/(nm)	d_33_/(pC·N^−1^)
362	52.6
725	69.4
1092	53.7

## Data Availability

Not applicable.
